# Supplementary material for: Reduced Abundance of Nitrate-Reducing Bacteria in the Oral Microbiota of Women with Future Preeclampsia
Source: Nutrients. 2022 Mar 8;14(6):1139. doi: 10.3390/nu14061139 (PMC8953404; doi:10.3390/nu14061139)
Supplement: Supplementary file 1 [file nutrients-14-01139-s001.zip › nutrients-1599466-supplementary.pdf]

Table S1. Primer sequences

Target	Forward primer sequence 5′ → 3′	Reverse primer sequence 5′ → 3′	Length of the primer product
V. dispar	GAAGGCGACTTTCTGGACGA	ACCACATACTCCACCGCTTG	230
V. parvula	AGCACTTTGGGTGGGAACTC	GTACGTGTGTAGCCCAGGTC	105
narH	ACGACATGGACAAAGTCGAAGA	GCGCATGTATTCACGCATGT	413
Total bacteria (16S rRNA gene V1-V9)	GCAGGCCTAACACATGCAAGTC	CTGCTGCCTCCCGTAGGAGT	1465
V6-V8 16S rRNA gene	TCGTCGGCAGCGTCAGATGTGTATAA GAGACAGAAACTYAAAKGAATTGRCGG	GTCTCGTGGGCTCGGAGATGTGTATAAG AGACAGACGGGCGGTGWGTRC	466

Table S2. Dietary intake data for participants at 28 weeks gestation.

Characteristic	DPE	Control	P-value
Number	12	24	
Portion Standard Factor	0.87 (0.7-1.0)	0.8 (0.75-1.0)	0.63
Energy (kJ/day)	5353 (4068-6822)	6103 (4938-7977)	0.28
Total Fat (g/day)	60.88 (45.0-81.88)	50.22 (36.22-73.38)	0.14
Saturated Fat (g/day)	28.2 (21.87-36.6)	25.4 (14.9-29.5)	0.25
Poly-saturated Fat (g/day)	7.4 (6.1-11.5)	4.6 (3.8-11.3)	0.16
Monosaturated Fat (g/day)	22.1 (16.4-28.9)	15.9 (12.5-26.0)	0.15
Protein (g/day)	69.5 (56.0-96.4)	61.9 (48.4-74.4)	0.22
Carbohydrate (g/day)	139.2 (113.1-191.6)	137.9 (116.3-170.2)	0.75
Sugars (g/day)	73.36 (56.5-93.7)	67.9 (53.7-84.0)	0.57
Starch (g/day)	64.4 (49.7-89.8)	77.5 (60.4-111.8)	0.25
Fiber (g/day)	14.5 (12.2-18.0)	17.1 (13.5-21.6)	0.26
Cholesterol (mg/day)	331.3 (199.3-383.8)	221.7 (114.8-278.8)	0.04
Potassium (mg/day)	1965 (1687-2490)	2128 (1817-2745)	0.29
Sodium (mg/day)	1870 (1589-2667)	1617 (1299-2227)	0.20

Data presented as the median with interquartile range
